# Supplementary material for: Children’s spatial language skills predict their verbal number skills: A longitudinal study
Source: PLoS One. 2022 Oct 31;17(10):e0277026. doi: 10.1371/journal.pone.0277026 (PMC9621456; doi:10.1371/journal.pone.0277026)
Supplement: S1 Table — (PDF) [file pone.0277026.s001.pdf]

S1 Table. Sequence of items, children's responses translated from German into English, and children's responses scored as correct in each case for the spatial language skills task.

Correct term	Responses	Coded as correct
In the box	In	In
	Inside/indoor	
	Blue box	
Under the table	Under	Under
	Under the bed	Under the bed
	Behind	
	In front of	
	In	
Behind the house	Behind	Behind
	In the house	
	On the side of the house	
	At the house	
	Under the house	
Between the flowers	Between	Between
	In the grass	
	At the flowers	
	Next to	
	Behind	
On the table	On	On
	On top of	On top of
	On the bed	On the bed
	Above	
In front of the car	In front of	In front of
	In	
	Under	
	On the street/ground	
	Next to	
	Behind	
Next to the flower	Next to	Next to
	On the grass	
	At the flower(s)	
	Behind	
	Between	
	In the center	
