# Supplementary material for: Children’s spatial language skills predict their verbal number skills: A longitudinal study
Source: PLoS One. 2022 Oct 31;17(10):e0277026. doi: 10.1371/journal.pone.0277026 (PMC9621456; doi:10.1371/journal.pone.0277026)
Supplement: Attachment — Submitted filename: Response to Reviewers.docx [file pone.0277026.s003.docx]

We thank the editor and the two reviewers for their positive assessment of our revision. We have taken Reviewer #1 advice into account in revising the manuscript.

**Reviewer #1:**

The authors have done a fantastic job in responding to all my questions. I appreciate that the authors have now included regression models in the result section. I also like the fact that they have discussed the low reliability of the visual-perception task and elaborated on the findings that some of the covariates did not correlate with verbal number skills and/or spatial language. I only have a small comment regarding the abstract. I would remove the sample size from the abstract as the main analysis regarding the effect of spatial language on verbal number skills was only conducted on 40 children (and not 75). Apart from that I have no further comments or suggestions and I believe that the study is suited for publication in PlosOne.

We thank Reviewer 1 for this advice and have removed the sample size from the abstract.

**Reviewer #2:**

The authors addressed all questions and comments of the reviewers. I believe this is a good contribution to the literature.
